# High‐Throughput Screening and Interpretable Machine Learning for Rational Design of Bimetallic Catalysts for Methane Activation

**DOI:** 10.1002/advs.202524394

**Published:** 2026-03-14

**Authors:** Mingzhang Pan, Tian Zhang, Jiawei Dong, Yubao Xie, Kaifeng Zhong, Wei Guan, Haiqiao Wei, Changcheng Fu, Yaqiong Su

**Affiliations:** ^1^ College of Mechanical Engineering Guangxi University Nanning China; ^2^ Guangxi Institute of Artificial Intelligence Guangxi University Nanning China; ^3^ State Key Laboratory of Engines Tianjin University Tianjin China; ^4^ School of Chemistry Engineering Research Center of Energy Storage Materials and Devices of Ministry of Education National Innovation Platform (Center) for Industry‐Education Integration of Energy Storage Technology Xi'an Jiaotong University Xi'an China

**Keywords:** bimetallic catalysts, density functional theory, high‐throughput screening, machine learning, particle swarm optimization

## Abstract

Methane's efficient catalytic removal is vital for sustainable development. Bimetallic catalysts, though promising for methane activation, pose a design challenge due to their complex compositional space. This work introduces an integrated framework that combines high‐throughput density functional theory (DFT) and interpretable machine learning to accelerate the rational design of catalysts. Computational screening of face‐centered‐cubic (FCC) bimetallic catalyst surfaces identifies the bond cleavage energies of the first and the second C─H bonds and methyl adsorption energy as a key descriptor governing successive C─H activation and the shift in the rate‐determining step (RDS). Through the synergistic interaction of these descriptors, machine learning models can be constructed more effectively, leading to the discovery of a bimetallic catalyst for consecutive C─H bond cleavages that outperforms conventional natural gas engine aftertreatment systems. Based on the computationally derived DFT dataset, four machine learning models were trained using a particle swarm optimisation (PSO) algorithm, from which the optimal model capable of accurately predicting C─H bond energies was selected. This model also further revealed the dominant electronic structural features of the predictive model through SHapley additive interpretability (SHAP) analysis. This work establishes an interpretable, data‐driven methodology for designing high‐efficiency multicomponent catalysts.

## Graphical abstract

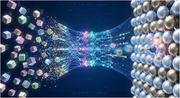

## 1 Introduction

Methane (CH_4_), the principal constituent of natural gas, is a potent greenhouse gas whose 100‐year global warming potential (GWP) is roughly 28 times that of CO_2_ [1, 2]. Concurrently, as the global natural‐gas vehicle fleet is projected to reach 35 million by 2030 [3], the carbon intensity of the transport sector has emerged as a decisive benchmark for national climate pledges. Hence, the discovery of a catalyst capable of ultra‐efficient abatement of methane in natural‐gas engine exhaust is paramount to attaining China's carbon‐neutrality ambition. [4, 5]

Bimetallic alloys composed of two transition metals (TMs) are regarded as among the most promising catalysts for methane activation owing to their vast compositional space and high intrinsic activity, rooted in concurrent geometric and electronic modulation [6, 7, 8, 9]. Conventional principles of surface science and microkinetic models (MKM) point out that the initial dissociative adsorption of methane (the first C─H bond cleavage) is the rate‐determining step (RDS) [10, 11] and obeys the Brønsted–Evans–Polanyi (BEP) linear relationship [11, 12, 13], However, advances in nanocatalysis, single‐atom alloys (SAAs), and bimetallic catalysts have generated mounting theoretical and experimental evidence indicating that [14, 15, 16, 17, 18], under defined conditions, the RDS shifts markedly from the first C─H bond cleavage to the dehydrogenation of surface methyl intermediates. In equimolar alloys, the surface exhibits an alternating arrangement of the two metallic atoms [19, 20], a distinctive geometric and electronic architecture that can restructure the reaction pathway and relocate the RDS to the cleavage of the second C─H bond [21], Once the catalyst overcomes the initial dual kinetic barriers of methane activation, multisite adsorption drives the reaction spontaneously toward deep dehydrogenation, culminating in complete methane oxidation [22, 23, 24], Consequently, the activation and cleavage of the first two C─H bonds emerge as the vital elementary steps guiding the screening of high‐performance catalysts. While complete methane oxidation entails a broader kinetic cascade involving oxygen activation and C‐O bond formation, overcoming the initial C─H kinetic barrier remains the universal descriptor for predicting catalytic efficiency [25, 26, 27].

Catalyst development has traditionally relied on trial‐and‐error approaches that are both time‐consuming and resource‐intensive [28, 29, 30], Density functional theory (DFT) has emerged as a powerful instrument for elucidating atomistic catalytic mechanisms, particularly for methane C─H bond activation. Recent advances have further demonstrated that the d‐band center model offers quantitative insight into adsorption energetics on TMs surfaces [31, 32, 33], Nevertheless, conventional DFT screening remains computationally prohibitive when the compositional space to be explored is vast. Machine learning has revolutionized materials discovery by establishing quantitative structure–property relationships; domain‐specific implementations in catalysis have already shown promise in predicting both activity and stability [34]. Shu et al. recently employed interpretable machine learning to decipher structure sensitivity in metallic catalysts, [35] whereas Xiao's team integrated data‐driven models with reaction phase diagrams to unravel oxide–molecule interactions for rational catalyst design [36], Yet, most existing studies still lack atomistic mechanistic insight into the RDS shift during methane oxidation [37], Current catalysis‐oriented machine‐learning models typically operate as “black boxes,” thereby hindering fundamental scientific understanding [38], The SHapley additive interpretability (SHAP) framework provides a rigorous theoretical basis for model interpretability, but its potential remains under‐exploited in complicated catalyst design [39], Moreover, although the performance of pure‐metal catalysts in methane oxidation has been extensively examined, systematic investigations of face‐centered‐cubic (FCC) structured bimetallic alloys with well‐defined dopant configurations are still scarce. The quantitative correlation between electronic‐structure descriptors (d‐band characteristics, Bader charges) and the successive energetics of C─H bond cleavage awaits further clarification.

This work establishes a multiscale framework that integrates SHAP‐interpretable machine learning with high‐throughput DFT to enable rational design of FCC‐structured 1:1 doped alloys for methane C─H bond activation. A systematic DFT campaign was conducted for 104 distinct FCC alloy configurations (8 FCC base metals by 13 dopants), explicitly locating transition states and calculating reaction and activity energies for the first two C─H bond‐cleavage elementary steps. By tracking the RDS shift across alloy catalysts, while incorporating the Sabatier principle [40], this study confirms that the CH_3_ adsorption energy constitutes the vital descriptor; only when this value falls within 0.13–0.43 eV might the activation barriers for both C─H cleavage events simultaneously reach their minima. After the high‐throughput DFT screening, IrPt emerges as the optimal alloy, exhibiting superior activity relative to the benchmark PdPd reference. Four regression algorithms—Random Forest (RF), Support Vector Regression (SVR), Multilayer Perceptron (MLP), and eXtreme‐Gradient Boosting (XGBoost)—were trained to predict the consecutive C─H bond cleavage energies; particle swarm optimization (PSO) was further embedded to enhance the predictive accuracy under the constraint of limited data. SHAP provides model interpretability and enables post‐validation, revealing the intrinsic relationship between electronic structural features and catalytic performance metrics. By employing this model, it is possible to predict and identify alloy catalysts with higher activity than those identified through DFT screening alone.

## 2 Results and Discussion

### 2.1 Element‐Dependent Trends in C─H Bond Cleavage Energetics

Eight FCC TMs—Ni, Cu, Rh, Pd, Ag, Ir, Pt, and Au—were Selected as Base Elements and Individually Doped with Thirteen Common Metals (Ni, Cu, Rh, Pd, Ag, Ir, Pt, Au, Ti, Mn, Fe, Co, and Zn) to Synthesize 104 Uniformly Distributed Catalysts (i.e., M_1_M_2_) with a 1:1 Stoichiometric Ratio on the (111) Surface, Comprising 96 Bimetallic Alloys and 8 Pure Metallic Structures. For each M_1_M_2_ Catalyst, the Surface Atom of the Base Element Was Designated as the Active Site for Methane Adsorption, and Comprehensive DFT Calculations Were Performed. Through Structural Optimization and Static Self‐consistent Calculations on Slabs, all Reaction Intermediates and Transition States Identified via Climbing Image Nudged Elastic Band (CI‐NEB), the Energy Characteristics of Intermediates and Transition States for each Catalyst, alongside Their Fundamental Physicochemical Properties, Geometric Features, and Electronic Structural Characteristics (Table S1). To Rationally Design Highly Active Catalysts Capable of Initiating Methane C─H Bond Activation, the Constructed FCC M_1_M_2_ Library Was Subjected to Integrated Thermodynamic and Kinetic Screening, and the Optimal Elementary‐step Sequence for the First Two C─H Bond Activations Was Established on the Basis of Literature Pathways [41]. (Figure S1). The BEP Principle is Widely Recognized as a Basic Rule of Heterogeneous Catalysis [42]; for Methane Oxidation, It Allows the Activation Barrier of the Crucial Elementary Step to be replaced by Its Reaction Energy, Enabling Rapid Catalyst down‐selection through Adsorption‐energy Calculations Instead of Costly Transition state Searches. Consequently, the Reaction Energy of the First and the Second C─H Bond Cleavages Was Adopted as the Activity Descriptor.

**FIGURE 1:**
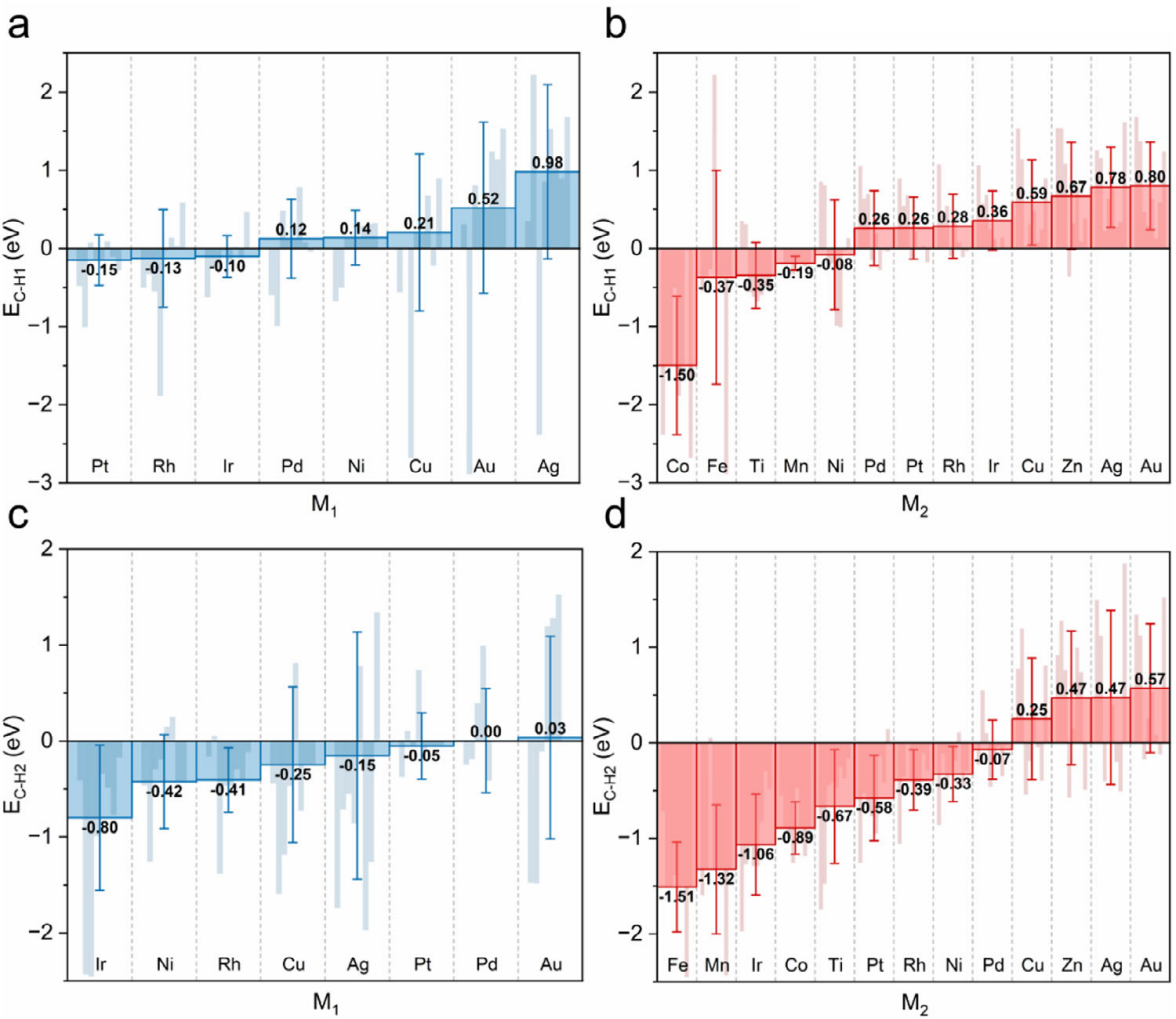
Energy of C─H bond cleavage (E_C─H1_ and E_C─H2_) for the first two C─H bonds on M_1_M_2_ catalysts. (a) Average E_C─H1_ values and error bars for each M_1_ site element (sample size n = 13 per bar). Data are presented as mean ± standard deviation (SD), where error bars represent the SD of the same data group, derived from distributions of different dopants (n = 13 dopants per M_1_ base metal). (b) Mean E_C─H1_ values and error bars for each M_2_ site element (sample size n = 8 per bar). (c) Mean E_C─H2_ values and error bars for each M_1_ site element (n = 13). d) Mean E_C─H2_ values and error bars for each M_2_ site element (n = 8).

According to the conventional BEP relationship, theoretically, a lower reaction energy implies a lower C─H bond activation energy barrier [43], which is typically more conducive to the complete oxidation of methane. The first C─H bond cleavage energies (E_C─H1_) of M_1_M_2_ were calculated using Equation S1. As shown in Figure 1a, E_C─H1_ across all systems exhibits a range from −0.15 to 0.98 eV. Among the eight base elements, Pt, Rh, and Ir exhibit lower E_C─H1_, with average E_C─H1_ values of ‐0.15, ‐0.13, and ‐0.10 eV, respectively. These elements have indeed been extensively studied and applied as commercial catalysts [44]. We selected the most used element, Pd, as the reference. The average E_C─H1_ values for the remaining four TMs were positive, ranging from 0.14 to 0.98 eV, indicating relatively weak methane activation capabilities. Conversely, we investigated the promotion effect of M_2_ on the first C─H bond cleavage of CH_4_ (Figure 1b). Surprisingly, Co exhibits a particularly pronounced enhancement of methane activation, with an average E_C─H1_ value of −1.50 eV. This indicates that the introduction of Co effectively modulates the electronic structure and surface properties of the primary active metal, significantly optimizing the alloy surface's adsorption capacity for CH_3_ molecules and H atoms. In contrast, when Cu, Zn, Ag, and Au as the dopant elements M_2_, the average E_C─H1_ values all exceeded 0.50 eV, rendering the initial activation thermodynamically unfavorable. Notably, examinations of Figure 1a,b reveal that E_C─H1_ values for Cu, Au, and Ag decreased significantly upon doping with partial metallic elements Co or Fe, highlighting the complexity inherent in studying the activity of alloy catalysts.

Figure 1c,d further elucidate the trends associated with another critical descriptor: the second C─H bond cleavage energy (E_C─H2_). As illustrated in Equation S2, E_C─H2_ correlates with the activation of the methyl species and involves the subsequent adsorption of the methyl molecule and a hydrogen atom. Notably, the distribution pattern of E_C─H2_ exhibits a distinct deviation from that of E_C─H1_. In contrast to the first C─H activation step, Ir is the lowest average E_C─H2_ value of ‐0.80 eV (Figure 1c). Corroborated by the results in Figure 1a, this indicates that Ir surfaces maximally stabilize the CH_3_ intermediate while weakening the remaining C─H bonds, rendering the second dehydrogenation step thermodynamically most favorable. Conversely, the average E_C─H2_ values for other substrate elements cluster within a narrow range of ‐0.42 to 0.03 eV, representing a significant reduction compared to E_C─H1_. Furthermore, observations from Figure 1d reveal that Fe and Mn at the doping site M_2_ are the most conducive to the E_C─H2_, exhibiting average E_C─H2_ values of ‐1.51 and ‐1.32 eV, respectively. Simultaneously, analogous to the first C─H bond cleavage process, the average E_C─H2_ values remain positive when Cu, Zn, Ag, and Au are employed as dopants M_2_. This suggests that when utilized as dopants, these elements render the alloy surface relatively weak in adsorbing both methyl and methylene species. Consequently, they are ill‐suited as active components for promoting deep oxidation of methane, particularly in reactions necessitating multi‐step dehydrogenation.

Upon closer scrutiny of Figure 1, it becomes evident that certain metal elements exhibit pronounced standard deviations. For instance, when Ag occupies the M_1_ site, the standard deviation of E_C─H2_ reaches as high as 1.29 eV (Figure 1c). This observation underscores that the impact of a specific element on the C─H bond cleavage energy does not follow a monolithic trend; rather, it is highly sensitive to the combinatorial effect of the substrate and dopant. We identify that elements manifest divergent adsorption capacities toward reaction intermediates depending on their role as either the base substrate or the dopant. Consequently, substantial performance volatility is observed even for the same element across varying alloy configurations. This electronic and geometric heterogeneity ultimately induces a migration of the RDS for methane oxidation among the critical elementary reactions.

### 2.2 RDS Shift and Catalyst Activity Screening

The Activation Energies (E_a1_ and E_a2_) of the First C─H Bond Cleavage and the Second C─H Bond Cleavage of M_1_M_2_ Were Calculated Using Equations S3 and S4. The Relationship between the Reaction Energy and Activation Energy for the First Two C─H Bond Cleavage Elementary Reactions Is Presented in Figure S2. Although the Overall Trend of the First C─H Bond Cleavage Largely Conformed to the BEP Relationship (Figure S2a), a Certain Deviation Emerged during the Second C─H Bond Cleavage Process (Figure S2b). These Deviations Are Particularly Pronounced in Certain Alloy Combinations. This Indicates That the Simple Descriptors E_C─H1_ and E_C─H2_ Derived from the BEP Relationship Are Insufficient to Fully Characterize the Successive C─H Bond Cleavage Dynamics on Bimetallic Surfaces. This Observation Is Consistent with Recent Studies Indicating That Methane Oxidation on complex catalysts involves multiple factors highly sensitive to Surface Composition [45]. This Underscores the Necessity for Systematic, Comprehensive Investigations of Bimetallic Alloys Incorporating Machine Learning, Particularly in 1:1‐doped FCC Alloys with Alternating Metal Atom Arrangements. Integrating Our Detailed Analyses of E_C─H1_ and E_C─H2_, We Observe a Close Correlation between the Adsorption Energy of Methyl Molecules and the Key Elementary Reactions in Methane Activation.

**FIGURE 2:**
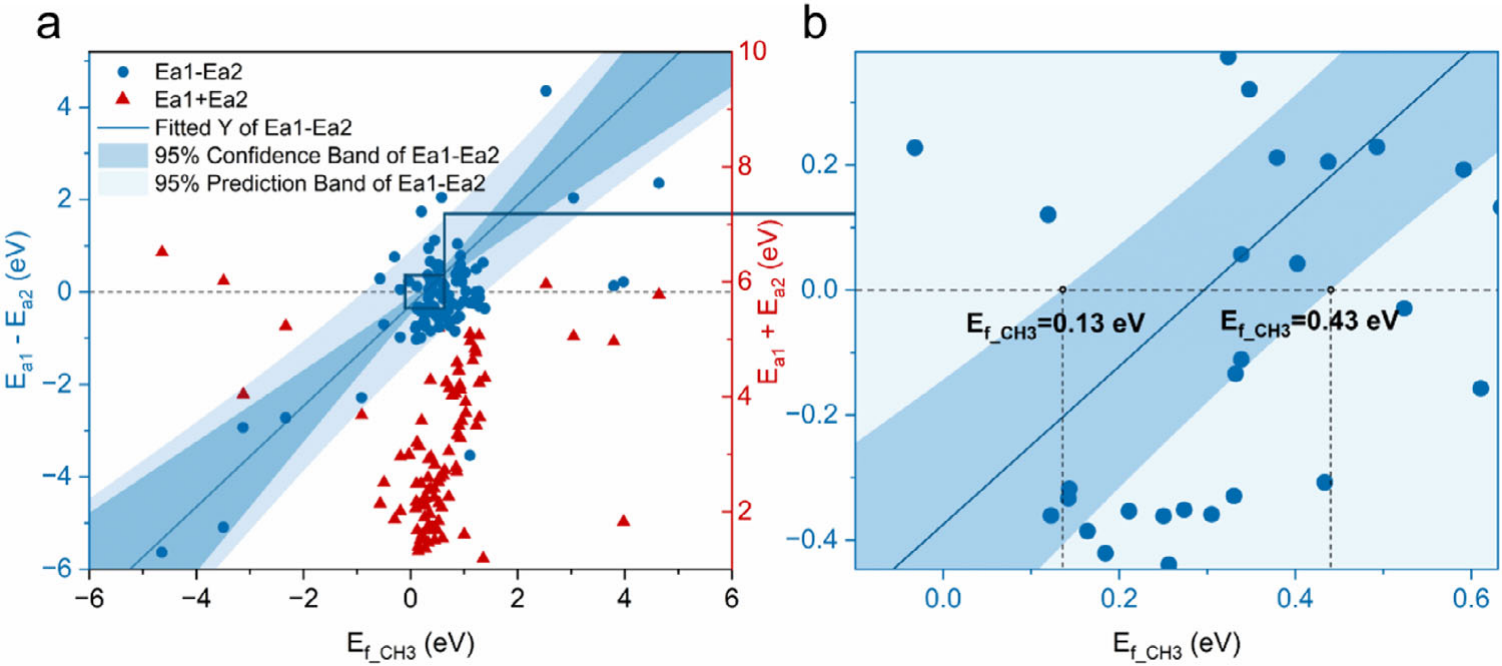
The relationship between methyl adsorption energy and RDS transfer. (a) The relationship between the sum and difference of the activation energies (E_a1_ and E_a2_) for the first and second C─H bond cleavages in M_1_M_2_ molecules, respectively, and the adsorption energy of the methyl molecule. b) The enlarged vision of the black rectangle in Figure 2a.

The Energies of Methyl Molecules (E_CH3_) and the Energies Required to Adsorb Methyl Molecules (E_f_CH3_) Were Calculated Respectively Using Equations S5 and S6. Figure 2a Presents the Correlation of E_f_CH3_ with both E_a1_‐E_a2_ and E_a1_+E_a2_. Symbols above the Reference Line (E_a1_‐E_a2_ = 0) Correspond to Catalysts Where the Barrier for the First C─H Cleavage Is Higher, Establishing that Step as the RDS. Conversely, Those below the Line Have a Higher Barrier for the Second C─H Cleavage, Making It the RDS. This Systematic Distribution Demonstrates That the RDS Shifts between the First and the Second C─H Bond Cleavages. The Sabatier Principle Is the Foundation for Understanding the Role of Methyl Adsorption Energy in the Two‐stage Reaction [46]. This Energy Must be neither Too Strong nor Too Weak; Its Magnitude Is Predicted to influence the Identity of the RDS. Figure 2a Reveals That Only Alloy Catalysts Exhibiting Moderate CH_3_ Adsorption Energies (approximately 0.13‐0.43 eV) Simultaneously Exhibit Markedly Suppressed Activation Barriers for both the First and the Second C─H Bond Cleavages. To evaluate the Statistical Reliability of the Descriptor‐based Relationship, We Provide both the 95% Confidence Band and the 95% Prediction Band in Figure 2a. The Confidence Band Quantifies the Uncertainty of the Fitted Trend, Whereas the Prediction Band Reflects the Expected Dispersion of Individual Data Points around the Fit. The Presence of Narrow and Consistent Intervals around the Identified Region Supports the Robustness of the Optimal E_f_CH3_ Window (0.13–0.43 eV) for Simultaneously Minimizing the Two Activation Barriers. This Observation Aligns Closely with the Sabatier Principle, Which Points out That Optimal Catalytic Activity Occurs at Intermediate Adsorption Strengths [47]. Consequently, E_f_CH3_ Is Established as a Pivotal Third Descriptor for Predicting the Activity of Bimetallic Methane‐oxidation Catalysts.

**FIGURE 3:**
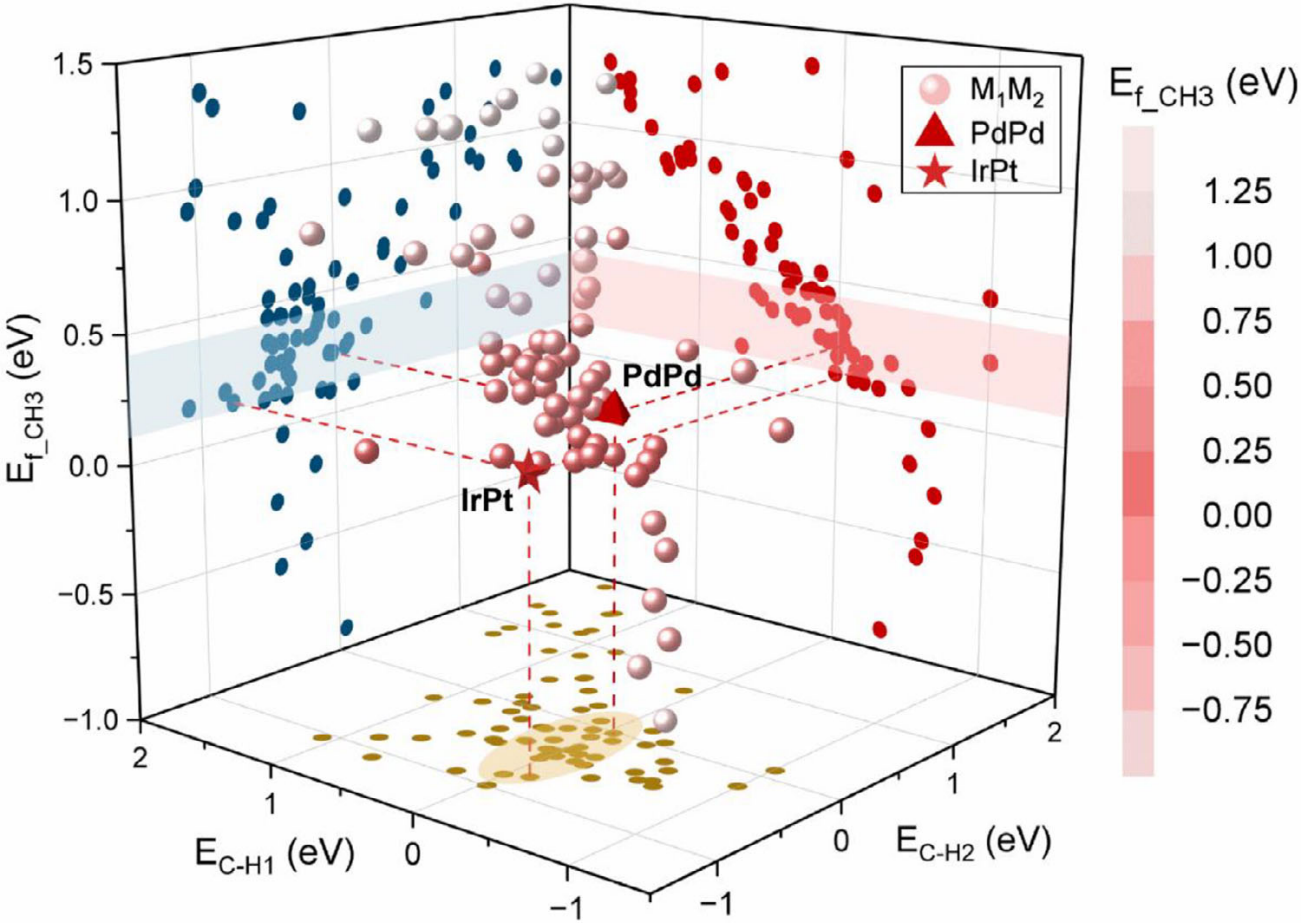
Activity prediction of alloy catalysts for methane oxidation via specific descriptors.

Building on the above insights, E_C─H1_, E_C─H2_, and E_f_CH3_ were selected as descriptors. PdPd, a pure‐metal catalyst that is frequently utilized in commercial applications and commonly adopted for total methane oxidation in the extant literature [48, 49, 50], served as the benchmark. As illustrated in Figure 3, the 3D scatter plot illustrates the distribution of 104 M_1_/M_2_ catalyst candidates based on three key descriptors: the first C─H bond cleavage energy (E_C−H1_, x‐axis), the second C─H bond cleavage energy (E_C−H2_, y‐axis), and the methyl adsorption energy (E_f_CH3_, z‐axis). Pink spheres represent the alloy library; the red triangle marks the conventional PdPd benchmark; the red star denotes the identified optimal IrPt alloy. The blue, red, and gold dots on the back and bottom planes represent 2D projections of the 3D data, highlighting the correlations between pairs of descriptors. The shaded bands (light blue and pink) along the E_f_CH3_ axis and the yellow‐shaded ellipse on the basal plane define the “golden zone” (moderate E_f_CH3_ between 0.13–0.43 eV, combined with minimized E_C−H1_ and E_C−H2_). Compared with the benchmark PdPd, these materials represent a range of promising high‐performance candidates. Among them, IrPt exhibits the lowest E_C─H1_ and E_C─H2_ values of ‐0.12 eV and ‐0.77 eV, which are markedly lower than those of the conventional Pd‐based reference (E_C─H1_ = ‐0.04 eV, E_C─H2_ = ‐0.02 eV). Preliminary analysis for IrPt identifies the cleavage of the second C─H bond as the RDS of the overall reaction.

**FIGURE 4:**
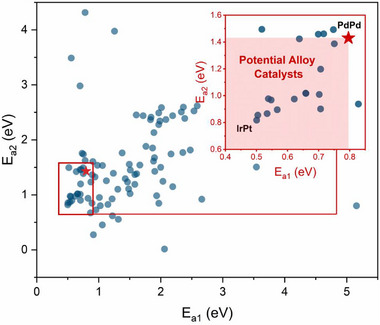
Verification of the methane oxidation activity of alloy catalysts using the activation energy calculated by DFT. The red diagram in the upper right is an enlarged version of the red rectangle in the lower left.

Figure 4 displays the DFT‐computed activation‐energy validation, wherein the catalytic activity is quantified directly by the barriers of the two elementary steps (E_a1_ and E_a2_). Using PdPd as a reference point, it is evident that only combinations falling within the lower‐left area satisfy the selection criterion. The enlarged inset in the upper‐right corner reveals that the red‐shaded area contains the optimal catalysts; notably, IrPt once again emerges as the leading candidate. The activation energies for sequential C─H bond cleavage on IrPt (E_a1_ = 0.50 eV, E_a2_ = 0.82 eV) are substantially lower than those on PdPd (E_a1_ = 0.80 eV and E_a2_ = 1.43 eV). These findings collectively validate the combined descriptors E_C─H1_, E_C─H2_, and E_f_CH3_ as reliable metrics for the preliminary identification of highly efficient methane‐oxidation catalysts.

**FIGURE 5:**
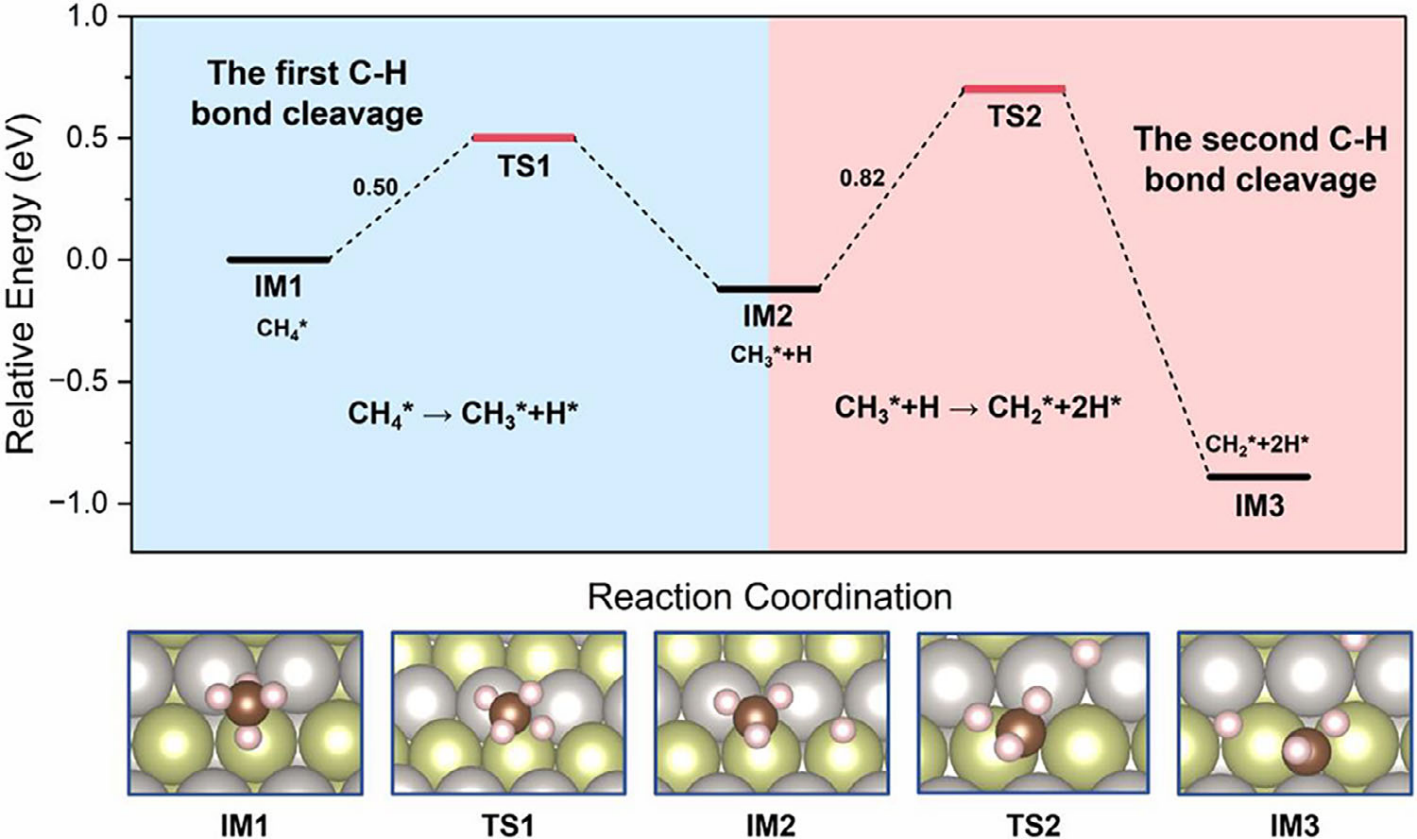
Reaction pathway of the key elementary steps in methane oxidation over IrPt. The structures of intermediates and transition state in the reaction cycle are shown below, where atoms with light green, grey, brown, and white represent Ir, Pt, C, and H, respectively.

The reaction pathway of the key elementary steps in methane oxidation [51] over the IrPt alloy is shown in Figure 5. The first C─H bond activation of methane occurs at an Ir active site (IM1 → IM2, E_a1_ = 0.50 eV). The resulting CH_3_^*^ remains adsorbed on the same Ir atom, whereas the liberated H^*^ binds to a surface hollow site before migrating. CH_3_^*^ then undergoes further dehydrogenation (IM2 → IM3, Ea2 = 0.82 eV), producing CH_2_^*^ that migrates from the Ir site to a three‐atom hollow site composed of the original Ir atom, a neighboring Ir atom, and the nearest Pt dopant.

As shown in Table S2 (Supplementary Information), the synergistic interaction between the base Ir atom and the Pt dopant is evidenced by the significantly enhanced adsorption energy of CH_2_^*^ on IrPt (E_f__CH2 = ‐0.09 eV) compared to pure Ir(111) (0.25 eV) and Pt(111) (0.80 eV). Bader charge analysis shows a charge redistribution between Ir and Pt, which modifies the d‐band characteristics of the active hollow site [52, 53, 54]. This electronic modulation, combined with the optimized geometric ensemble of the Ir‐Ir‐Pt site, lowers the energy of the CH_2_^*^ intermediate relative to its predecessors in the reaction pathway. Such stabilization is crucial for facilitating the deep dehydrogenation of methane by effectively lowering the energy of the subsequent transition states, thereby validating the exceptional capability of IrPt for total methane oxidation [51, 55, 56].

### 2.3 Exploration of Autonomous Optimization Model of Interpretable

Conventional catalyst development relies heavily on trial‐and‐error protocols and expert intuition, incurring protracted experimental cycles, prohibitive testing costs, and an intrinsically limited search space. A synergistic framework that couples high‐throughput first‐principles calculations with machine learning can efficiently mine the deep features embedded in theoretical data and construct regression models endowed with autonomous experience, thereby accelerating the discovery and optimization of high‐performance catalysts. This paradigm constitutes a pivotal step toward overcoming the entrenched bottlenecks of high cost and heavy human dependence [57]. Establishing a high‐fidelity materials database and navigating the expansive, multidimensional hyper‐parameter landscape are indispensable prerequisites for trustworthy regression; however, the vast number of adjustable hyper‐parameters in machine‐learning regressors renders exhaustive manual tuning prohibitively laborious and rarely yields truly optimal configurations.

To simultaneously address the dual challenges of catalyst screening and hyper‐parameter optimization, this research proposes a regression framework that integrates PSO. Systematic construction and comparative evaluation of four machine‐learning algorithms revealed that the PSO‐embedded approach outperforms conventional manual tuning [58], enabling the rational design of methane‐oxidation alloy catalysts. SHAP, a game‐theoretic explainability framework, was employed to interpret the predictive “black‐box” model [59]. The integration of these strategies establishes an interpretable machine‐learning framework that ultimately uncovers the latent rules linking input features to catalytic performance.

Spearman correlation analysis is prioritized due to its core advantage of evaluating monotonic relationships between variables rather than being confined to linear associations [60, 61, 62]. Unlike Pearson's correlation coefficient based on raw observed values [63, 64, 65], Spearman's correlation coefficient is calculated using ranks, making it insensitive to outliers and capable of effectively identifying non‐linear yet monotonic intrinsic correlations. Among the 104 bimetallic alloy surface systems covered in this study, key electronic descriptors, such as d‐band centers and Bader charge, exhibit consistent (monotonic) yet not necessarily linear regulation of C─H bond cleavage energy (E_C─H_). This property enables the Spearman coefficient, based on rank evaluation, to accurately capture such nonlinear trends, thereby avoiding the underestimation or misjudgment of highly predictive descriptors caused by the linearity assumption of Pearson correlation. Furthermore, the downstream prediction model XGBoost, as a gradient‐boosted decision tree algorithm, determines its splitting thresholds based on feature value ranking [66]. This mechanism is highly aligned with the rank‐based nature of the Spearman coefficient. Accordingly, employing Spearman correlation for feature selection ensures methodological alignment between descriptor selection criteria and the core algorithm's operational mechanism. This guarantees that selected descriptors provide effective splitting nodes for XGBoost decision trees, thereby enhancing modeling efficiency and prediction robustness.

**FIGURE 6:**
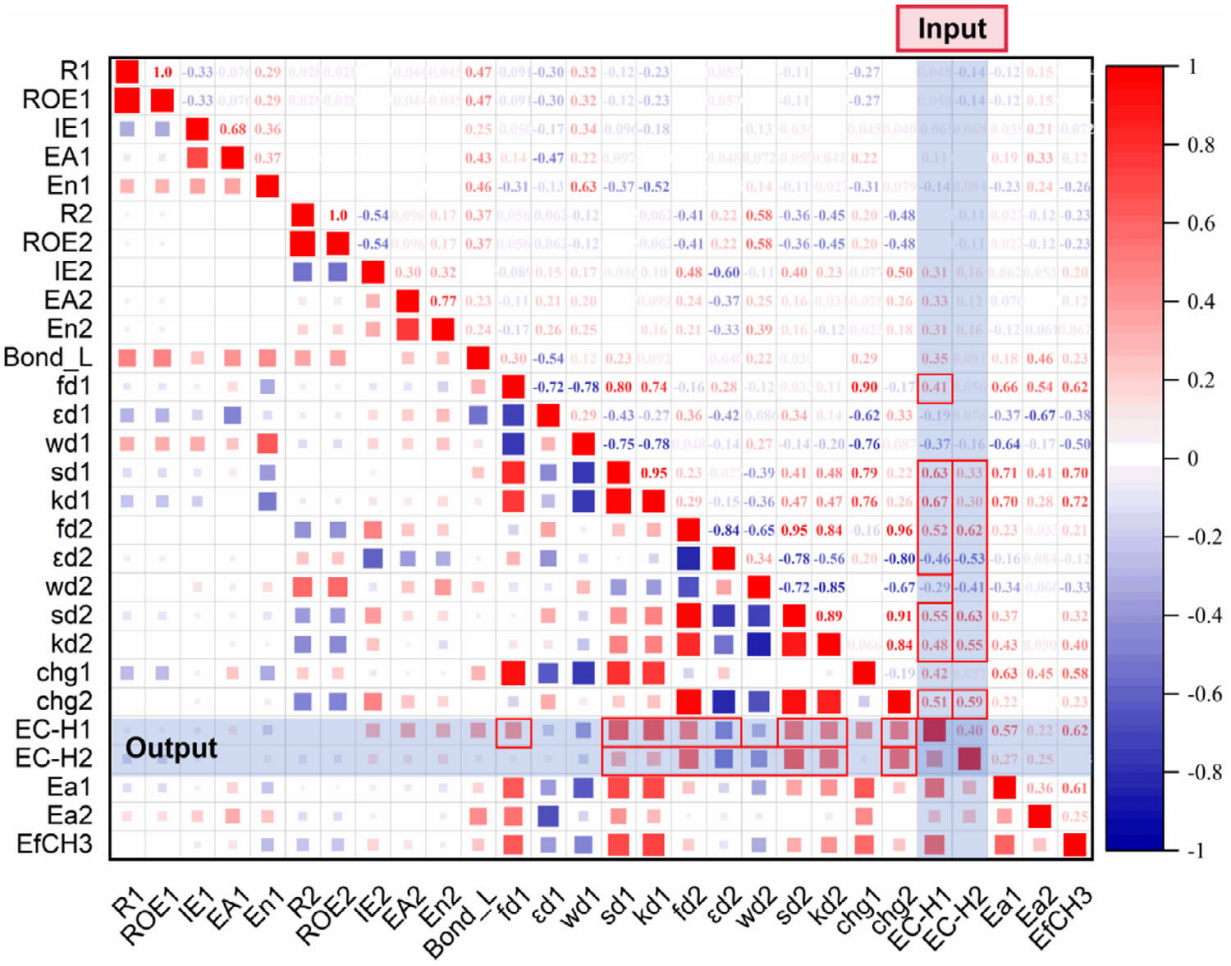
The spearman correlation matrix of all structural features and reaction properties. The red and blue colours indicate positive and negative correlations between variables. (The size of the rectangle is directly proportional to the magnitude of the numerical value.).

To uncover the intrinsic physical drivers of catalysis, we established a comprehensive feature space encompassing both geometric strain (e.g., metal bond lengths Bond_L, atomic radii R_1_/R_2_) and electronic effects (e.g., Bader charges, d‐band characteristics). Spearman correlation analysis (Figure 6) reveals that purely geometric descriptors exert only a marginal direct influence on the energies of the first and the second C─H bonds in methane. In contrast, surface d‐band characteristics and Bader charges (representing charge transfer) have a markedly stronger effect. This observation aligns with the Nørskov d‐band theory, suggesting that geometric strain primarily influences adsorption energetics indirectly by altering the overlap of metallic d‐bands (electronic structure) [67]. Consequently, electronic descriptors serve as more direct and effective predictors. Previous studies have established a pronounced positive correlation between C─H bond energy and activation energy. When the catalyst satisfies E_f_CH3_ within a defined window, a lower C─H bond energy corresponds to a lower activation energy and thus higher catalytic activity. Consequently, E_C─H1_ and E_C─H2_ were selected as the model outputs, and the eight descriptors exhibiting the strongest correlation with each C─H bond energy were adopted as inputs.

**FIGURE 7:**
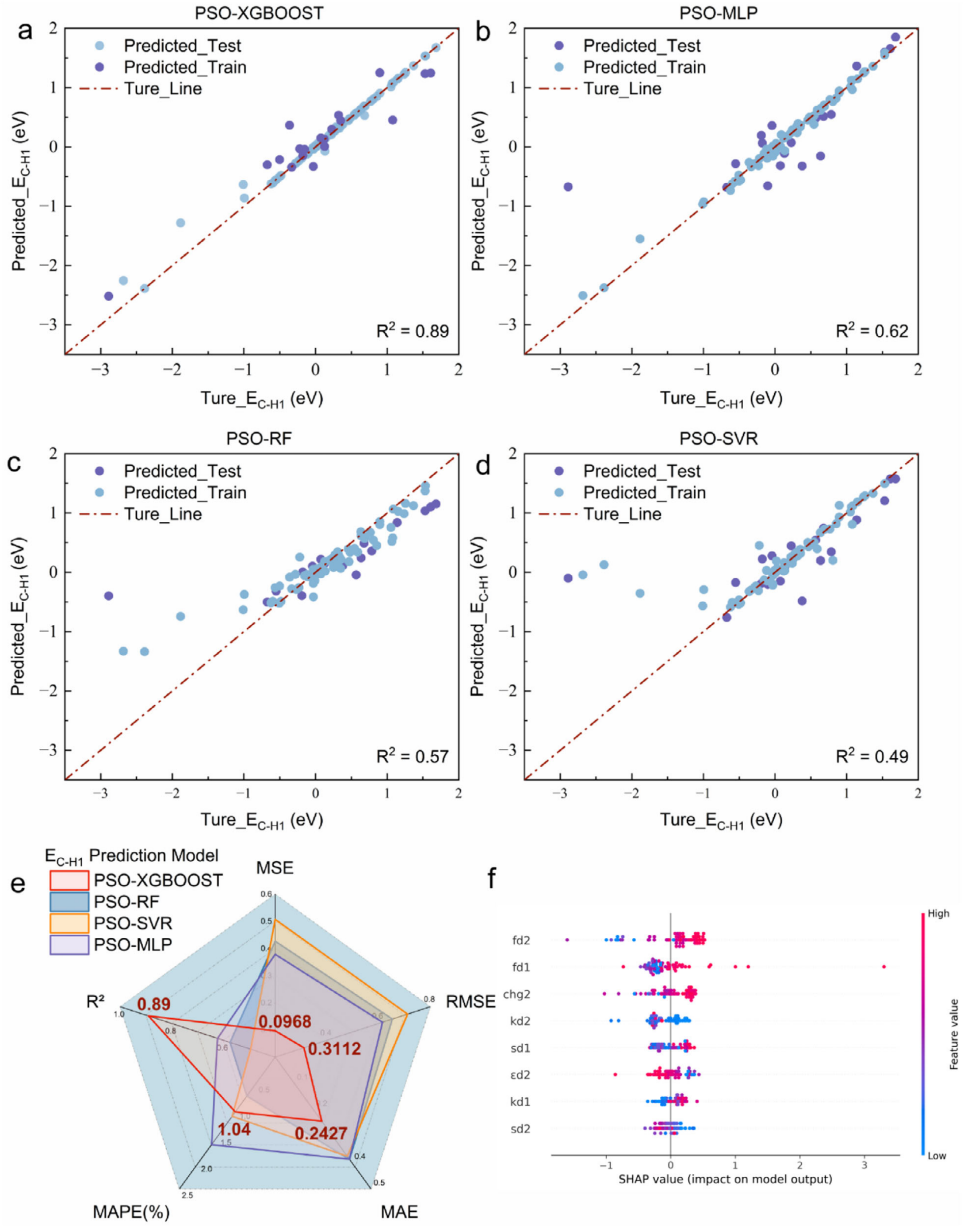
Four machine learning models respectively predict the bond energy of the first C─H bond cleavage and analyze the evaluation indicators. (a), (b), (c), and (d) correspond to the training and prediction results of the four machine learning models: PSO‐XGBOOST, PSO‐MLP, PSO‐RF, and PSO‐SVR, respectively. (e) Comparison of five evaluation metrics across the four machine learning models. The PSO‐XGBoost model achieves the best performance with R^2^ = 0.89. (f) SHAP model interpretability analysis of the optimal prediction model, demonstrating how each input variable specifically influences the output result.

**FIGURE 8:**
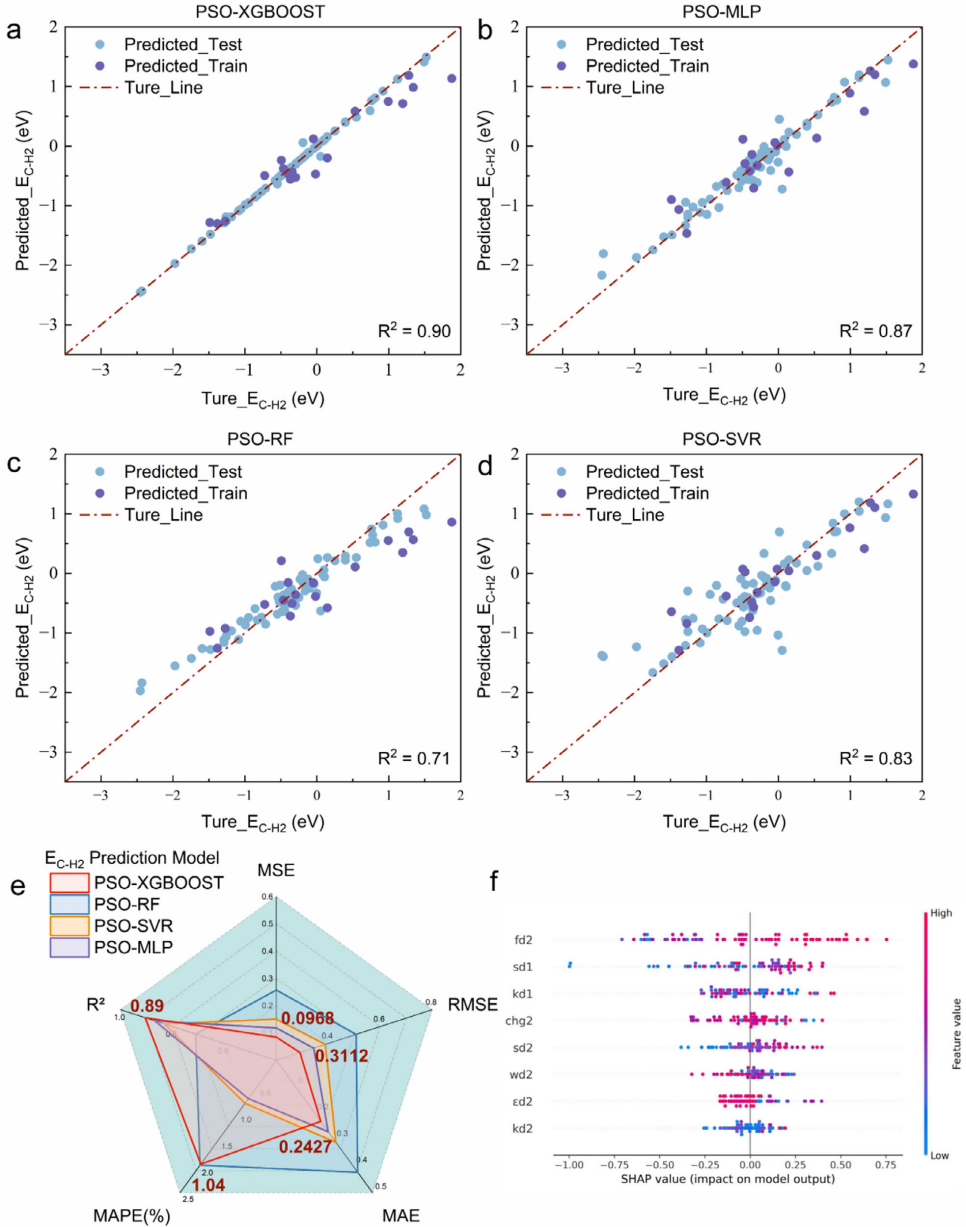
Four machine learning models respectively predict the bond energy of the second C─H bond cleavage and analyze the evaluation indicators. (a), b), (c), and (d) correspond to the training and prediction results of the four machine learning models: PSO‐XGBOOST, PSO‐MLP, PSO‐RF, and PSO‐SVR, respectively. (e) Comparison of five evaluation metrics across the four machine learning models. The PSO‐XGBoost model achieves the best performance with R^2^ = 0.89. (f) SHAP model interpretability analysis of the optimal prediction model, demonstrating how each input variable specifically influences the output result.

This study systematically compared four machine‐learning algorithms, XGBoost, RF, SVR, and MLP, and employed PSO to navigate the high‐dimensional hyper‐parameter landscape of each learner. Model performance was rigorously evaluated on the test set through Mean Squared Error (MSE), Root‐Mean‐Squared Error (RMSE), Mean Absolute Error (MAE), Mean Absolute Percentage Error (MAPE) and the coefficient of determination (R^2^). These comprehensive metrics serve as a quantitative assessment of the reliability and generalization of the machine‐learning predictions, ensuring the robustness of the developed models even under limited‐data regimes. As illustrated in Figures 7 and 8, PSO‐XGBoost better uncovers the underlying patterns in the data than its competitors, thereby achieving superior predictive accuracy. For the E_C─H1_ task (Figure 7e), PSO‐XGBoost (MSE = 0.0968, RMSE = 0.311, MAE = 0.243, MAPE = 1.04%, R^2^ = 0.89) outperforms all alternative models across every metric. In the E_C─H2_ task (Figure 8e), PSO‐XGBoost (MSE = 0.0847, RMSE = 0.291, MAE = 0.231, MAPE = 1.96%, R^2^ = 0.90) registers a marginally higher MAPE than PSO‐MLP and PSO‐SVR, yet the ensemble of the remaining four indicators confirms its superior overall fit. The PSO configuration and the optimized XGBoost hyper‐parameters are listed in Table 1. To achieve an improved trade‐off between global exploration and local exploitation, the PSO algorithm adopted herein incorporates a dynamic inertia‐weight strategy throughout the iterative process. Specifically, the inertia weight decreases linearly with successive iterations, prioritizing extensive global search during the early phase while concentrating on the refinement of local optima in the later stage.

**TABLE 1:** Hyper‐parameter setting for PSO Algorithms and XGBoost Predictive Model Training.

Algorithm/Model	Hyper‐parameter	Value	Hyper‐parameter	Value
PSO	particles	300	iterations	200
	Individual learning factor	1.50	Social learning factor	1.50
	w_max	0.90	w_min	0.40
	W(Inertia weight) = w_max – (w_max – w_min) × (iteration / n_iterations)			
XGBOOST/EC─H1	n_estimators	716	max_depth	9
	learning_rate	0.28	subsample	0.53
	colsample_bytree	0.55	gamma	0.56
	reg_alpha	5.25	reg_lambda	8.59
	max_delta_step	6	scale_pos_weight	4.02
XGBOOST/EC─H2	n_estimators	696	max_depth	9
	learning_rate	0.25	subsample	0.74
	colsample_bytree	0.99	gamma	1.35
	reg_alpha	2.09	reg_lambda	5.39
	max_delta_step	2	scale_pos_weight	2.84

A critical challenge in applying machine learning to atomistic catalysis is the risk of the “curse of dimensionality” and subsequent overfitting when dealing with limited high‐fidelity DFT data. While contemporary deep learning frameworks typically require vast data arrays to converge implicitly, recent epistemological advances in data‐driven catalysis verify that tree‐based ensemble methodologies can achieve remarkable robustness and generalizability on small‐sample datasets [68, 69, 70], provided the input features encode profound physical intuition. In this work, the apparent limitation of the 92‐sample dataset is overwhelmingly compensated by the extraordinary information density of the 28 engineered descriptors. By expanding the feature space beyond the conventional d‐band center to include full higher‐order d‐band moments (width, skewness, kurtosis) and atomic Bader charges, the descriptors act as a robust physical inductive bias. Furthermore, the XGBoost algorithm fundamentally resists overfitting through its rigorous internal L_1_ and L_2_ leaf‐weight regularizations. The integration of PSO to autonomously tune hyperparameter depth and learning rates effectively prevented the model from memorizing localized numerical noise.

To further benchmark the predictive power of our PSO‐XGBoost framework, we compared it with the Random Forest (RF) algorithm, a state‐of‐the‐art method frequently employed in recent computational catalysis studies. As shown in Table S3, under identical descriptors and PSO optimization, the RF model only yielded an R^2^ of 0.443–0.579, significantly underperforming compared to our model.

Based on the SHAP analysis (Figures 7f and 8f), the individual contribution of each input feature to the C─H bond energy predicted by the PSO‐XGBOOST model is quantitatively disclosed. The results reveal that the d‐band filling of the doped element (f_d2_) serves as the most influential common descriptor for both E_C─H1_ and E_C─H2_ predictions. Physically, the f_d2_ value dictates the occupancy of the d‐orbitals in the dopant atom, which fundamentally modulates the density of states at the active site. This electronic feature directly governs the strength of the interaction between the metal surface d‐electrons and the antibonding orbitals of the C─H bonds, thereby determining the activation barriers [71, 72, 73]. In addition, the SHAP summary highlights the importance of other pivotal descriptors—such as the Bader charge of the surface dopant (chg_2_) and residual d‐band characteristics [74, 75, 76]. Specifically, the Bader charge (chg_2_) serves as a proxy for the ligand effect, reflecting the charge redistribution between the base metal and the dopant. This cooperatively modulates the local chemical environment and electrostatic potential of the active site, collectively determining the adsorption stability of key intermediates (e.g., CH_3_) and the resulting C─H bond energy.

### 2.4 Conclusion

High‐throughput DFT calculations were employed to conduct a systematic investigation of 1:1‐doped FCC alloy catalysts, re‐examining the RDS mechanism of methane oxidation on bimetallic surfaces. Using the reaction energies of the first two C─H bond activations as primary descriptors and the methyl adsorption energy as a supplementary descriptor, IrPt was identified from 104 candidates as a high‐performance catalyst for methane activation. It is demonstrated that, on the 1:1 alloy surface, the key elementary steps of methane conversion cannot be rationalized by conventional linear relations. By integrating SHAP‐based explainable machine learning techniques with the PSO optimization algorithm, precise predictions of catalytic descriptors were achieved under limited dataset conditions, revealing that the “electronic‐structure characteristics” of the alloy atoms, particularly the d‐band filling and Bader charge of the dopant, exert a decisive influence on overall activity. Furthermore, the electronic structure of the alloy atoms is a key factor that determines when the RDS changes. Although this study primarily focuses on intrinsic activity screening, the established framework is highly extensible; future research could incorporate deactivation‐related descriptors, such as carbon diffusion barriers and metal‐surface binding energies, to develop high‐fidelity models for predicting catalyst stability under realistic operating conditions. Combining systematic DFT screening with interpretable machine learning, this study provides rational design principles for efficient diatomic alloy catalysts in natural‐gas engine after‐treatment and establishes a foundation for future advances in the field.

Furthermore, the dataset, comprising 104 configurations, was intentionally restricted to the FCC (111) facet and a 1:1 stoichiometric ratio to establish a controlled baseline for extracting fundamental electronic descriptors. While sufficient for small‐dataset interpretable machine learning, real‐world catalysts exhibit higher morphological complexity. Future investigations must leverage transfer learning and expanded DFT campaigns to evaluate varied M_1_/M_2_ ratios and higher‐index facets.

## 3 Methods

### 3.1 DFT Calculations and Database Construction

First‐principles Calculations Were Conducted Using the Vienna Ab Initio Simulation Package (VASP) [77, 78] to evaluate the Energetics (adsorption, reaction energetics, and activation barriers) of CH_4_ Decomposition over Different Catalysts. The Projector Increased Wave (PAW) [79, 80], Potentials Were Employed for Electron‐ion Interaction, and the Generalized Gradient Approximation (GGA) Method with the Perdew, Burke, and Ernzerhof (PBE) [81], Functional Was Used for Exchange and Correlation Potential [82], A Monkhorst–Pack Mesh with (3 × 3 × 1) k‐points and a Plane‐wave Energy Cutoff of 400 eV Was Applied to the Four‐layer p‐(4 × 4) metallic Surfaces. The Top Two Layers of these Metallic Surfaces Were Allowed to Relax to Facilitate Their Electronic Interactions with the Adsorbates, While the Bottom Two Layers Were Fixed at Their Bulk Positions. The Configurations Reached Optimization When the Electronic Energy Converged to 10^−^^5^ eV and the Ionic Forces Fell below 0.05 eV Å^−^^1^ [83]. CI‐NEB Was Used for Locating Transition States (TS), and Stretching Frequencies Were Analyzed to Identify a TS with Only One Imaginary Frequency [84, 85].

The initial computational screening matrix was systematically constructed using 8 FCC base transition metals (Ni, Cu, Rh, Pd, Ag, Ir, Pt, and Au) and 13 common metallic dopants, yielding a comprehensive set of 104 theoretical M_1_M_2_ (1:1 stoichiometric) configurations. However, to ensure the absolute fidelity of the subsequent machine learning models and to eliminate computational artifacts, rigorous data curation protocols were applied post‐DFT relaxation. During the structural optimization and CI‐NEB transition state searches, 12 configurations were strictly excluded from the final analytical dataset. These exclusions comprised instances of non‐convergent electronic self‐consistent field (SCF) calculations arising from extreme lattice mismatch, as well as configurations undergoing massive surface reconstructions that irreversibly obliterated the defined 1:1 alternating active site geometry. Consequently, the final noise‐reduced dataset utilized for machine learning consisted of 92 pristine configurations.

### 3.2 Machine Learning Database Construction

The dataset for machine learning models comprises geometric structural properties, electronic structural characteristics, and energy features calculated via DFT, encompassing 96 bimetallic surface catalysts in a 1:1 ratio and 8 pure metal surfaces. The model training set is composed of 80% randomly selected from all the data, while the test set consists of the remaining 20% of the data. The characterization of bimetallic surfaces in machine learning models (i.e., model input features) encompasses both the fundamental and electronic properties of alloy components, with a particular focus on electronic properties such as D‐state distribution features (fill degree, center position, peak width, skew, and kurtosis). Detailed information on the input characteristics and symbols of bimetallic surfaces can be found in Supplementary Materials Table 1.

### 3.3 Interpretable Machine Learning

To construct predictive models, we tested a variety of machine learning algorithms, including EXtreme Gradient Boosting (XGBoost), Random Forest (RF), Support Vector Regression (SVR), and Multi‐layer Perceptron (MLP). The training data consists of randomly selected datasets of 1:1 and 80% of pure metal surfaces. This training dataset is further refined by eliminating outliers to ensure consistency within the 95th percentile of the data. The test set consists of the remaining 20% of the data. The model performance is evaluated by the mean square error (MSE), root mean square error (RMSE), mean absolute error (MAE), mean absolute percentage error (MAPE), and coefficient of determination (R2) of the test set. Moreover, the PSO algorithm is introduced for efficient hyperparameter optimization, breaking through the limitations of traditional manual search [86].

Understanding the rationale behind machine learning predictions is essential for trust, comprehension, and fairness. SHAP values [87] facilitate this understanding by quantifying the contribution of each feature to the prediction in complex models, such as RFs. SHAP values are based on Shapley values from cooperative game theory, reflecting the weighted average impact of a feature across all possible combinations of features. For a model with a prediction function f(x) over M features [88], the SHAP value for the ith feature, denoted as φi, is determined using the following formula:

(1) $$\phi_{i}=\sum_{S\subseteq M/\{i\}}\frac{|S|!(|M|-|S|-1)!}{|M|!}\;\left[f_{x}(S_{\cup }\{i\})-f_{x}\left(S\right)\right],$$

where M represents the total set of features, S is a subset of features excluding the ith feature, |S| denotes the size of subset S, fx (S_∪_ {i}) is the predicted value when the ith feature is included alongside S, and f_x_ (S) is the prediction for the subset S alone [11]. This equation explains how SHAP values allocate the variance from the mean prediction across different features, ensuring the fulfillment of paradigms like fairness, equivalence, non‐participating element, and cumulative effect.

### 3.4 Statistical Analysis

Data pre‐processing involved Min‐Max normalization for feature scaling and an outlier detection procedure based on the 95th percentile of the C─H bond cleavage energies (E_C─H_) [89], which reduced the bimetallic dataset from 104 to 92 samples to ensure physical consistency. Continuous data in comparative plots are presented as mean ± standard deviation (SD), where SD reflects the dispersion of catalytic properties across the compositional space. Sample sizes were n = 104 for the initial DFT screening, refined to n = 92 for machine learning, and further split into training (n = 74) and testing (n = 18) sets. Model performance was evaluated using R^2^, RMSE, and MAE. All statistical analyses and model training were conducted using Python 3.8.10 packages: Scikit‐learn (v1.0.2), XGBoost (v1.6.1), and SHAP (v0.41.0). DFT calculations utilized VASP 5.4.4.

## Conflicts of Interest

The authors declare no conflicts of interest.

## Supporting information




**Supporting File**: advs74837‐sup‐0001‐SuppMat.pdf.

## Data Availability

The data that support the findings of this study are available in the supplementary material of this article.
