# Supplementary material for: High‐Throughput Screening and Interpretable Machine Learning for Rational Design of Bimetallic Catalysts for Methane Activation
Source: Adv Sci (Weinh). 2026 Mar 14:e24394. Online ahead of print. doi: 10.1002/advs.202524394 (PMC13325640; doi:10.1002/advs.202524394)
Supplement: Supplementary file 1 — Supporting File: advs74837‐sup‐0001‐SuppMat.pdf. [file ADVS-9999-e24394-s001.pdf]

Supporting Information

High-Throughput Screening and Interpretable Machine Learning for Rational Design of Bimetallic Catalysts for Methane Activation

Mingzhang Pan[1,2]*, Tian Zhang[1], Jiawei Dong[1], Yubao Xie[1], Kaifeng Zhong[1], Wei Guan1*, Haiqiao Wei[3]*, Changcheng Fu[1], Yaqiong Su[4]*

1. College of Mechanical Engineering, Guangxi University, Nanning 530004, China

2. Guangxi Institute of Artificial Intelligence, Guangxi University, Nanning 530004, China

3. State Key Laboratory of Engines, Tianjin University, Tianjin 300072, China

4. School of Chemistry, Engineering Research Center of Energy Storage Materials and Devices of Ministry of Education, National Innovation Platform (Center) for Industry-Education Integration of Energy Storage Technology, Xi'an Jiaotong University, Xi'an, 710049, China

*Email: pmz@gxu.edu.cn; guanwei@gxu.edu.cn; whq@tju.edu.cn; yqsu1989@xjtu.edu.cn

1. Computational details

The C-H bond cleavage energies ($E_{C\text{-}H1}$ and $E_{C\text{-}H2}$) over M_1M_2 were calculated using equation S1 and S2, where $E_{CH_3{}^*+H^*}$, $E_{CH_4{}^*}$, $E_{CH_2{}^*+H^*}$ and $E_{CH_3{}^*}$ are the energies of adsorbed methyl and hydrogen after the first C-H bond activation, adsorption of methane molecules, adsorbed methylene and hydrogen after the second C-H bond activation and adsorption of methyl molecules. The activation energy (E_{a1} and E_{a2}) of the C-H bond cleavage in the first two steps were calculated using equation S3 and S4, where E_{TS1} and E_{TS2} are the energies of the transition state of the first C-H bond cleavage and the transition state of the second C-H bond cleavage. The energy of a methyl molecule (E_{CH_3}) were calculated using equation S5, where E_{CH_4} and E_{H_2} are the energies of gaseous methane molecules and hydrogen molecules. The energies

required to adsorb methyl molecules (E_{f_CH3}) were calculated using equation S6, where E_* is the energies of M_1M_2 slab.

$$E_{C\text{-}H1}=E_{CH_3^*+H^*}-E_{CH_4^*} \quad \text{(S1)}$$

$$E_{C\text{-}H2}=E_{CH_2^*+H^*}-E_{CH_3^*} \quad \text{(S2)}$$

$$E_{a1}=E_{TS1}-E_{CH_4^*} \quad \text{(S3)}$$

$$E_{a2}=E_{TS2}-E_{CH_3^*} \quad \text{(S4)}$$

$$E_{CH_3}=E_{CH_4}-1/2E_{H_2} \quad \text{(S5)}$$

$$E_{f\_CH_3}=E_{CH_3^*}-E_*-E_{CH_3} \quad \text{(S6)}$$

The adsorption energies of the CH_2* intermediate state were calculated using equations S7–S9, analogous to the previous formulae. Employing equations S7–S9 and energies obtained via DFT calculations, we determined the adsorption energies of the CH_2* intermediate state on IrPt, Ir, and Pt (111) surfaces, as shown in Table S2 (Supplementary Information).

$$E_{H^*}=E_{CH_3^*+H^*}-E_{CH_3^*} \quad \text{(S7)}$$

$$E_{CH_2^*}=E_{CH_2^*+H^*}-E_{H^*} \quad \text{(S8)}$$

$$E_{f\_CH_2}=E_{CH_2^*}-E_*-E_{CH_2} \quad \text{(S9)}$$

2. Supplementary figures

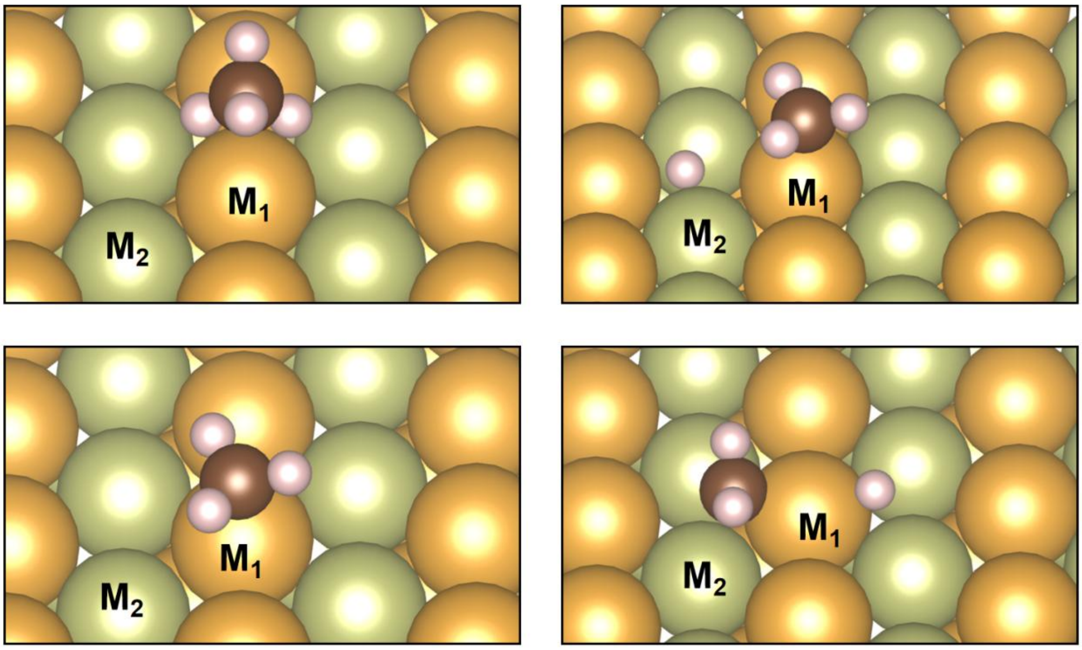

Figure S1. Methane activation step on M_1M_2. Atoms are presented using yellow (Au), green (Ir), brown (C), and white (H).

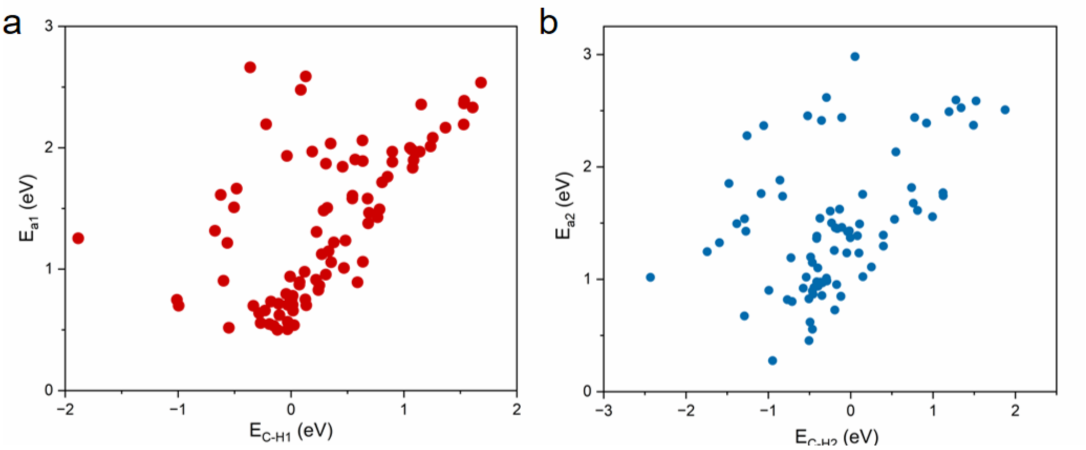

Figure S2. The BEP relationship of key elementary reactions

3. Supplementary tables

Table S1. Features for machine-learning models considered in this work.

**Feature value name**	**Meaning**
R_1	Atomic radius of M_1 (active site) atom

R_{OE1}	Orbital Extension of M_1 atom
IE_1	Ionization energy of M_1 atom
EA_1	Electron affinity of M_1 atom
En_1	Electronegativity of M_1 atom
R_2	Atomic radius of M_2 (base) atom
R_{OE2}	Orbital Extension of M_2 atom
IE_2	Ionization energy of M_2 atom
EA_2	Electron affinity of M_2 atom
En_2	Electronegativity of M_2 atom
Bond_L	Distance between M_1 and M_2
f_{d1}	Filling of d-band of M_1 atom
ε_{d1}	d-band center of M_1 atom
w_{d1}	Width of d-band of M_1 atom
s_{d1}	Skewness of d-band of M_1 atom
k_{d1}	Kurtosis of d-band of M_1 atom
f_{d2}	Filling of d-band of M_2 atom
ε_{d2}	d-band center of M_2 atom
w_{d2}	Width of d-band of M_2 atom
s_{d2}	Skewness of d-band of M_2 atom
k_{d2}	Kurtosis of d-band of M_2 atom
chg_1	Bader charge of M_1 atom
chg_2	Bader charge of M_2 atom

$E_{C\text{-}H1}$	The bond energy of the first C-H bond breaking
$E_{C\text{-}H2}$	The bond energy of the second C-H bond breaking
E_{a1}	The activation energy of the first C-H bond break
E_{a2}	The activation energy of the second C-H bond break
E_{f_CH3}	Adsorption energy of methyl molecules

Table S2. The adsorption energies of the CH_2* intermediate state on IrPt, Ir, and Pt (111) surfaces

**Name**	**E_{f_CH2} (eV)**
Ir	0.25
Pt	0.80
IrPt	-0.09

Table S3. The predictive accuracy and computational time for the first and second C-H bond cleavage energies across the three models

**Task**		**$E_{C\text{-}H1}$**	**$E_{C\text{-}H2}$**
**PSO-XGBoost (This work)**	R^2	0.89	0.9
	Test time [s]	*0.0022*	*0.0023*
PSO-RF	R^2	*0.525*	*0.599*
	Test time [s]	*0.0062*	*0.0051*
RF (Baseline)	R^2	*0.443*	*0.579*
	Test time [s]	0.0094	*0.0091*
